# Enabling Micro Injection Moulding Using a Soft Tooling Process Chain with Inserts Made of Mortar Material

**DOI:** 10.3390/mi12080857

**Published:** 2021-07-22

**Authors:** Kilian Krüger, Martin Kain, Yang Zhang, David Bue Pedersen, Matteo Calaon, Guido Tosello, Hans Nørgaard Hansen

**Affiliations:** 1 Department of Mechanical Engineering, Technical University of Denmark, Produktionstorvet, Building 427, 2800 Kongens Lyngby, Denmark; mkain@mek.dtu.dk (M.K.); yazh@mek.dtu.dk (Y.Z.); dbpe@mek.dtu.dk (D.B.P.); mcal@mek.dtu.dk (M.C.); guto@mek.dtu.dk (G.T.); 2 Department of Mechanical Engineering, Technical University of Denmark, Niels Koppels Allé, Building 404, 2800 Kongens Lyngby, Denmark; hnha@mek.dtu.dk

**Keywords:** micro injection moulding, soft tooling, hard tooling, tool steel, mortar, concrete, alumina, QR code, wear, thermal conductivity

## Abstract

The manufacturing of inserts for micro injection moulding made of mortar material is presented in this work. The fabrication of the mortar insert described in this publication relied on a versatile and relatively fast rapid prototyping process based on soft tooling. The mortar insert has a QR code with micro features on its surface, which was replicated in acrylonitrile butadiene styrene (ABS) polymer by the micro injection moulding process. With this approach, it is possible to fabricate hard inserts for micro injection moulding purposes that are able to compete with conventional-made inserts made of tool steel.

## 1. Introduction and Motivation

**Figure 1:**
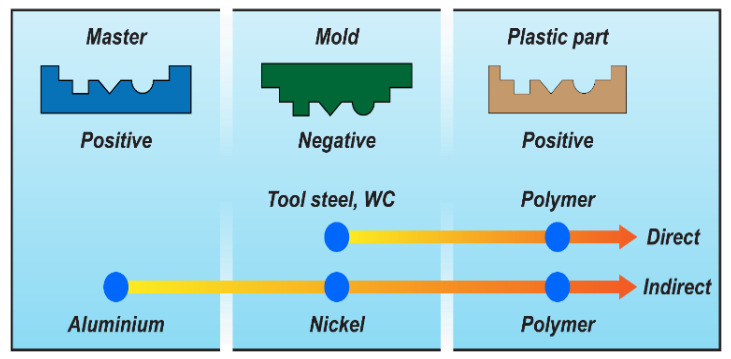
Examples of a direct and indirect tooling process chain. Reproduced with permission from [3].

The use of replication technologies for micro manufacturing has evolved over the last 10–15 years [1,2]. One of the main components of the replication process chain is the tool, which contains the negative geometry of the part, i.e., cavities on the final part are protrusions in the tool. The process chains available to create tools for micro injection moulding can be based on either a direct or indirect approach [3]. In the direct approach, the final tool geometry is fabricated directly—for example, by means of machining processes. In the indirect approach, a number of steps is employed whereby the geometry is mirrored (see Figure 1).

Additive manufacturing opens up new possibilities for creating geometries in the indirect tooling process chain. The concept of soft tooling using additive manufacturing has been reported in [4,5,6]. Soft tool inserts made by additive manufacturing are characterised by lower hardness, lower tensile strength, and a considerably lower modulus of elasticity than conventional metal tools. In addition, the thermomechanical loading during injection moulding stresses polymer tool inserts significantly, resulting in a shorter lifetime. On the contrary, the lead time of soft tools may be significantly reduced, and the flexibility of the additive manufacturing process chain can result in economic benefits [7].

Concrete and mortar are wildly used as materials for construction purposes worldwide [8]. Although this technology has been extensively investigated for decades, no published scientific work exists on the application of concrete or mortar for the fabrication of tools for injection moulding purposes, according to the best of the authors’ knowledge. This paper describes how mortar can be applied as a tool material in a soft tooling process chain for micro injection moulding. The experimental work was focused on documenting the ability of mortar to replicate micro features of a master geometry. It was also demonstrated how a soft master geometry, for example made by additive manufacturing, can form the starting point of the soft tooling process chain.

The test geometry for this study was a micro-milled QR code, which had been transferred to the surface of the mortar insert by casting in a soft mould. Then, the surface of the mortar insert was transferred to acrylonitrile butadiene styrene (ABS) polymer by micro injection moulding.

## 2. Mortar as a Tool Material

Mortar and concrete consist of a binder (often Portland cement clinker), water, and aggregates (often sand and gravel). A freshly mixed batch hardens over time because the binder and water react and form a solid. Additives may be used in small fractions to adjust the properties of the fresh or hardened material [8].

With the biggest aggregate grains smaller than 4 mm in diameter, the term mortar is usually used, while the term concrete is commonly used if aggregate grains bigger than 4 mm in diameter are incorporated in the mixture [9,10]. Since finely grinded aggregates were used in the study, the term mortar was employed in this article.

The mechanical properties of hardened mortar have several similarities to tool steel and fulfil some of the requirements addressed to tool steel for the fabrication of micro injection moulds: high strength and elastic modulus as well as high wear resistance and hardness. On the other hand, there may be some disadvantages of hardened mortar: poor ductility (brittleness) and thus poor toughness, bad machinability, and low thermal conductivity. However, making injection tools of mortar material in a fast and relatively cheap process may have cost benefits over conventional injection mould making [11,12].

**Figure 2:**
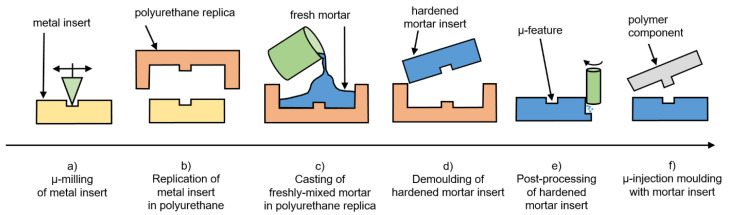
Soft tooling process chain.

The soft tooling process chain employed in this work consists of the following steps (Figure 2):

(a) Manufacturing the QR code geometry in steel by micro milling;
(b) Replication of QR code geometry by polyurethane (PU) casting;
(c) Casting of mortar in the soft polyurethane replica obtained in step (b);
(d) Separation of mortar from soft polyurethane replica (upon solidification of mortar);
(e) Fit and finishing of mortar insert to modular tool for injection moulding;
(f) Injection moulding of polymer component using mortar tool insert.

Process step (a) resulted in a metal insert, which acted as the benchmark insert. The mortar insert generated in process step (e) was compared against the metal (benchmark) insert.

However, it is not generally necessary to start with a metal insert. Alternatively, the casting form for the mortar insert, which was manufactured in process step (b), may be fabricated directly by additive manufacturing. This would open the opportunity for a process chain for the manufacturing of (micro) injection moulding inserts that relies neither on conventional tool making nor on employment of tool steel.

**Figure 3:**
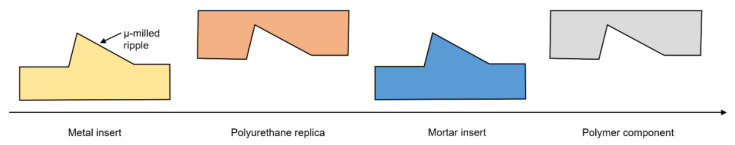
Mirroring of geometry throughout process steps.

The mirroring of the geometry throughout these process steps is explained based on a single micro-milled structure (ripple) of the QR code (Figure 3): QR code in steel (negative)—PU replica (positive)—mortar (negative)—polymer (positive). Due to the characteristics of the QR code/ripple geometry, the mirroring effect is not critical. However, should other geometries be considered for this soft tooling process chain, attention must be made to ensure that the desired geometry is achieved with the injection moulding process step.

## 3. Materials and Methods

### 3.1. Metal Insert with QR Code

**Figure 4:**
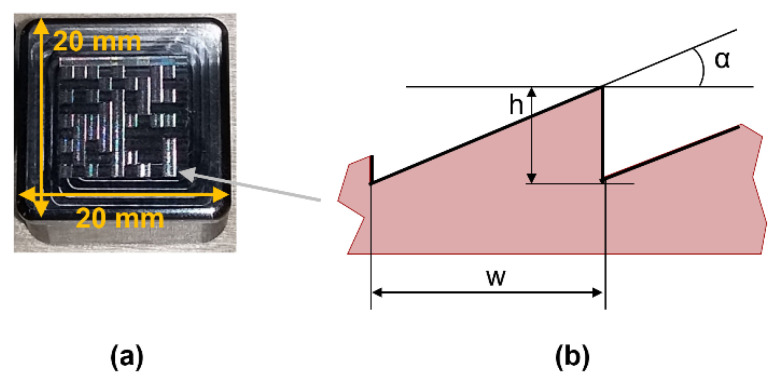
(**a**) Metal insert with 10 × 10 pixels on its surface; (**b**) Schematic profile of a micro-milled ripple with tilting angle α, ripple height h and ripple width w.

The experiment was based on an existing metal insert (length: 20 mm, width: 20 mm, thickness: 11 mm) with micro-milled features on the moulding side, forming the imprint of a QR code. The metal insert was made of tool steel (Orvar Supreme, Uddeholm/voestalpine HPM Denmark A/S, Kolding, Denmark). Fabrication of the metal insert and the QR code had been described in detail in [13]. Each of the 10 × 10 pixels of the QR code (Figure 4a) consists of several micro-milled ripples (Figure 4b).

### 3.2. Fabrication of Mortar Insert

The metal insert was replicated in polyurethane resin (Neukadur 8015, shore-hardness A80, Altropol Kunststoff GmbH, Stockelsdorf, Germany). This polyurethane replica acted as a casting form for freshly mixed mortar.

First, 150 g of Portland cement (PortlandWhite, Aalborg Portland A/S, Aalborg, Denmark) and 50 g of microsilica (Microsilica 971, Elkem ASA, Oslo, Norway) were mixed with 44.7 g of tap water and 14.7 g of superplasticiser (MasterGlenium ACE 460, Master Builders Solutions Deutschland GmbH, Trostberg, Germany in a vacuum mixer (Reitel Retomix Mini/400 mL bowl, Reitel Feinwerktechnik GmbH, Bad Essen, Germany) at 400 rpm for 2 min. Then, 250 g of Tabular Alumina (Tabular Alumina T 60/64, 0–0.5 mm, Almatis B.V., Rotterdam, The Netherlands) was added to the mixture, which was mixed for another 3 min.

The mixture was filled in the polyurethane casting form and hardened in a closed box at 100% relative humidity over 3 days at 20 °C and 1 day at 75 °C. The hardened mortar cast was peeled off the polyurethane casting form and externally adjusted using abrasive paper to fit in the injection mould cavity.

### 3.3. Micro Injection Moulding Experiment

**Figure 5:**
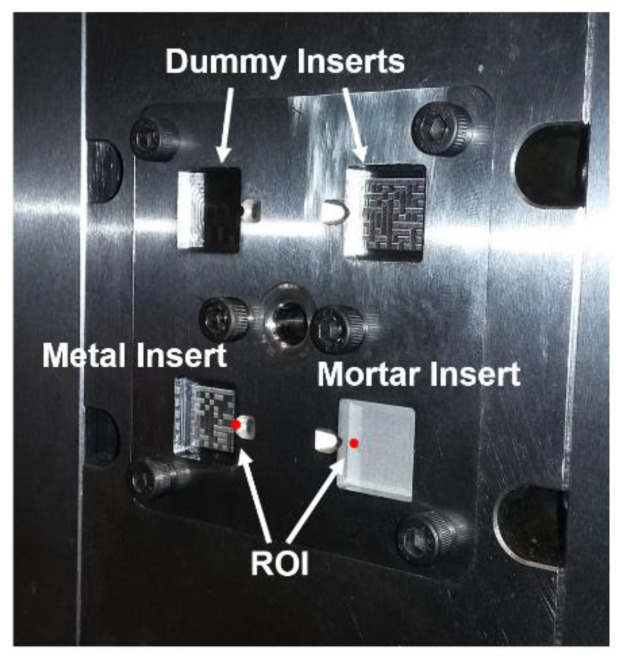
Metal insert and mortar insert being mounted on the injection mould. Picture taken prior to injection moulding. ROI: region of interest. Each of the four cavities has nominal dimensions of 12 mm × 12 mm.

The used micro injection mould had four equal part cavities for inserts. The first cavity was fitted with the mortar insert, the second cavity was equipped with the original metal insert, and the other cavities were filled with dummy inserts, as shown in Figure 5.

A micro injection moulding series of 1010 shots was performed on an injection moulding machine (Arburg A-370, 18 mm screw, Arburg GmbH + Co KG, Loßburg, Germany) with ABS polymer (Terluran GP-35, INEOS Styrolution Group GmbH, Frankfurt am Main, Germany), which was pre-dried with hot air at 80 °C for 4 h.

Process parameters were adapted from previous experiments with metal inserts only: the melt temperature of ABS was 230 °C (nominal value) and the mould was temperature-controlled to 50 °C (nominal value). Switch-over from the filling phase to the packing phase was triggered by the position of the screw, which was chosen such that each of the four cavities was approximately 98% full in the moment of switch-over. The screw injection speed was set to a constant of 100 mm/s. Packing pressure was set to constant 500 bar and packing time was set to 1.5 s. Cooling time was 10 s and the overall cycle time was 16.2 s.

### 3.4. Characterisation of Specimens and Data Analysis

The three-dimensional height map of the micro ripples was observed by laser scanning digital microscopy (Olympus LEXT OLS4100, Olympus Corporation, Shinjuku City, Tokyo, Japan). The specimens were inspected at 20 times magnification with an inspection area of 643 µm by 643 µm and a resolution of 1024 by 1024 pixels.

Data analysis of microscopy images was performed with SPIP—Analytical Software for Microscopy, Image Metrology A/S, Hørsholm, Denmark. Each three-dimensional height map was corrected for the tilting error (levelled by plane). No other corrections were applied to the raw data underlying a microcopy image.

The measurand under investigation was the tilting angle α. It was estimated based on a two-dimensional height profile, which had been extracted as the mean of the corrected and aligned three-dimensional height map.

This procedure secured that estimated tilting angle α:

- Was robust against noisy height data.
- Was robust against a potential offset error of measured height.
- Could be calculated easily based on the two-dimensional height profile.
- Did not depend on hard-to-measure single points (such as ripple height and ripple width).

## 4. Results and Discussion

**Figure 6:**
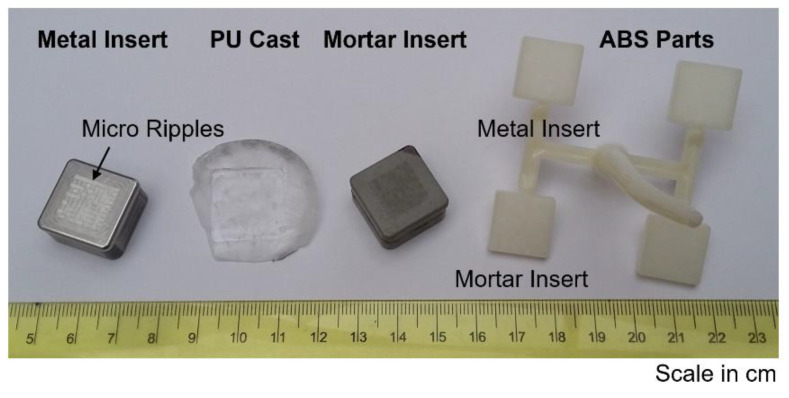
Products of mortar insert rapid prototyping process chain. The polyurethane (PU) replica is transparent. The acrylonitrile butadiene styrene (ABS) plastic component made with both inserts and by micro injection moulding is also shown.

In Figure 6, all products of the soft tooling process chain of the mortar insert are shown.

The injection moulding process parameters relating to the melt were selected based on the material data sheet provided by the material manufacturer. Other parameters were selected based on the requirements by the specific injection moulding tool. The packing time was defined by gate freezing, which could be attributed to design of the injection mould. Cooling time was defined by bending of the sprue, which was the part of the ABS component that cooled down the slowest.

**Figure 7:**
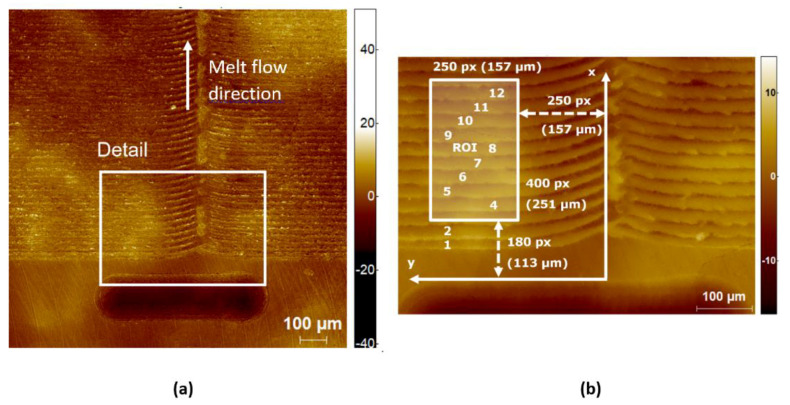
Corrected height map of QR code on mortar insert. (**a**) Overview, 10× magnification, 1278 µm × 1278 µm, 1024 pixels × 1024 pixels, z-scale in µm, z-range: 92 µm, height levelled by first-order polynomial; (**b**) Detail with region of interest, 20-fold magnification, 463 µm × 643 µm, 738 pixels × 1024 pixels, z-scale in µm, z-range: 31 µm, height levelled by first-order polynomial. The numbers 1 to 12 refer to different ripples.

Figure 7 shows the corrected height map of the area in focus as obtained with the confocal microscope. The identification of the analysed ridges is indicated in Figure 7b. The exact position of the region of interest is indicated by the coordinate system. With this identification, it is possible to find the same region of interest on all physical parts of the soft tooling process chain. On specimens with negative geometry (metal insert, mortar insert), the y-axis points to the left-hand side, whereas it points to the right-hand side on specimens with positive geometry (PU replica, ABS parts). This is due to the mirror effect, which comes along with each replication step.

**Figure 8:**
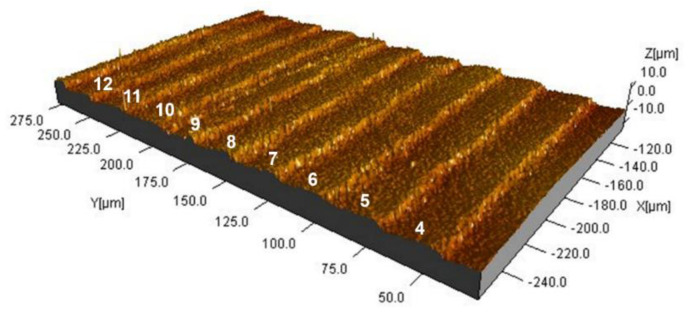
Corrected height map of region of interest of the QR code on the mortar insert. The height map was levelled by first-order polynomial in order to correct it for any tilting angle of the specimen. No other corrections were applied to the data underlying this figure. The numbers 4 through 12 refer to different ripples.

**Figure 9:**
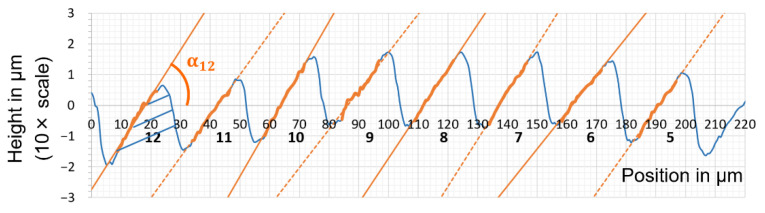
Mean height profile corresponding to Figure 8. The orange line estimated by linear regression and the x-axis were used for calculation of the eight tilting angles, respectively.

Figure 8 shows the selected region of interest from Figure 7. The ridges are clearly identifiable, and a certain degree of homogeneity is visible. It can be observed that there is a certain height variation of the ripples, and therefore, it was decided to calculate the tilting angle α of ripples 5 to 12, respectively (Figure 9).

**Figure 10:**
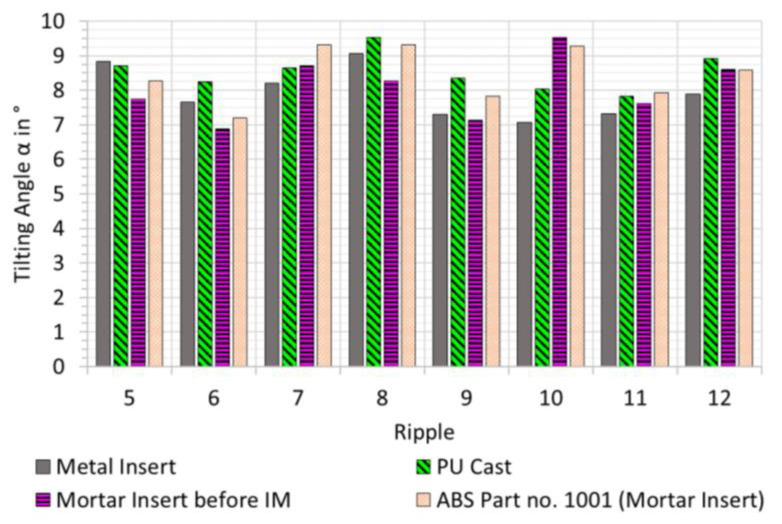
Tilting angle α for ripples 5 to 12 measured on metal insert, polyurethane cast (PU cast), and mortar insert, respectively, before injection moulding experiments had been performed. In addition and for comparison, the tilting angle measured on ABS part no. 1001 (moulded on mortar insert) is also included.

The evaluation of the corresponding ripple angles had been performed for every element under investigation: The steel insert, the PU cast, the mortar insert, and the resulting ABS part. Figure 10 shows the comparison of tilting angle of ripples 5 to 12 for each element, respectively. It can be observed that the angle of one specific ridge typically is within 1–1.5° between the four process steps. Ripple 10 is an exception to this. The PU cast generally seems to have steeper angles than the metal insert. Tilting angle seems to decrease from PU cast to the mortar insert, with the exception of ripples 7 and 10. From independent analysis of the functionality of the QR codes, we have seen that any variations of ridge angle above 5° will influence the functionality [14].

**Figure 11:**
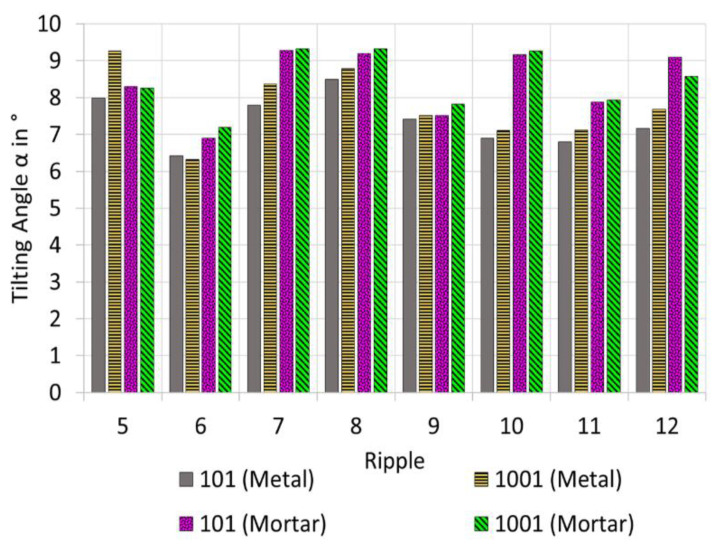
Tilting angles α measured on ABS parts micro injection moulded either with metal or a with mortar insert. The ABS components under investigation were shot no. 101 and no. 1001.

The injection moulding experiment had been conducted. The system was allowed 100 injection shots to stabilise. Figure 11 shows the comparison of ABS component no. 101 with ABS component no. 1001. In general, the change in the tilting angle for both metal and mortar inserts is typically below 1°. There seems to be an exception at ridge no 5. It has not been possible to conclude why the metal insert exhibits an increase in tilting angle. The experienced deviations could be a result of a measurement outlier, which may be due to contaminants on a particular ripple.

**Figure 12:**
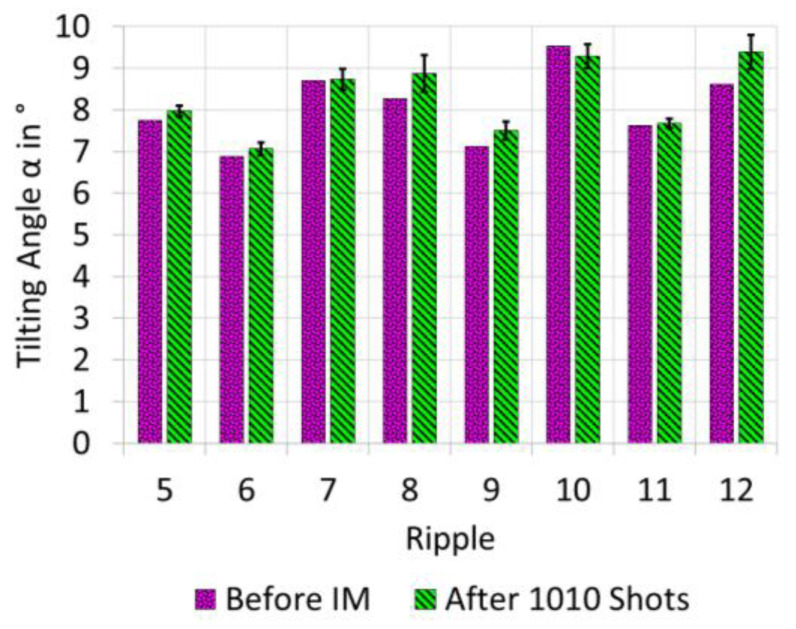
Tilting angle measured on mortar insert before injection moulding and after 1010 injection-moulded parts. The 95% confidence interval for repeatability of measurement is indicated individually by a black line for each ripple.

If wear occurs, it would be natural to expect a decrease in the tilting angle. Therefore, the tilting angle of the ripples on the mortar insert before injection moulding and after 1010 shots was considered (Figure 12).

The mortar insert after 1010 injection moulding experiments was used in order to quantify the repeatability of measurement: The height map was measured six times, and the tilting angle for each of the eight ripples and each of the six measurements was calculated. The confidence interval for repeatability of measurement was estimated for each of the eight ripples under the assumption of normal distribution of measurement results, for a confidence level of 95%, based on a degree of freedom of *ν* = 6 − 1 = 5 and thus with a coverage factor of *k* = 2.57. The 95% confidence interval of repeatability is in the range of 0.2–0.8°, depending on the ripple assessed.

We assume that—for a given ripple—the confidence interval for repeatability of tilting angle measurement does not vary from investigated sample to investigated sample (mortar insert before injection moulding/after 1010 shots), since it characterises the method under the influence of the operator rather than the specimen under observation. Based on this assumption, we could explain the difference in tilting angle with repeatability of tilting angle measurement only (overlapping confidence intervals). This means wear (if there was any) was sufficiently small compared to the repeatability of tilting angle measurement.

Therefore, it may be speculated here that the wear (rate) of mortar inserts used for injection moulding might be very low. The number of shots should be increased by one order of magnitude (10,000 shots) or even two orders of magnitudes (100,000 shots) for effective wear to occur. More studies and in-depth analysis have to be conducted in order to assess the lifetime of mortar inserts. Thorough and strict statistical tests will have to be applied on repeated experiments/measurements with numerous mortar inserts in order to detect and quantify wear (rate) of mortar material. Additionally, other contributions to measurement uncertainty have to be considered in order to evaluate wear in detail.

These observations with respect to wear may be assigned to the high content of hard and thus wear-resistant Tabular Alumina filler in particular. Cutting mortar inserts with corundum blades and even diamond blades was hardly possible, which strengthens this interpretation.

There was no observation of cracking of the mortar insert during the injection moulding experiment. ABS did not stick to the surface of the mortar insert, and the demoulding of ABS did not cause any problems. All three replication steps (metal insert to polyurethane cast to mortar insert to ABS component) combined did not lead to any significant surface defects with respect to the measurand under observation and the methods used.

Based on the evaluation of the tilting angle of micro features moulded from tool steel and mortar inserts, it can be concluded that the cycle time of 16.2 s was sufficient in both cases, although there is a notable difference in thermal conductivity between mortar and tool steel. For Orvar Supreme, the thermal conductivity at 20 °C is $25\frac{W}{m\cdot K}$ [15], whereas it is typically $3\frac{W}{m\cdot K}$ for dry concrete with improved thermal conductivity [16].

On the one hand, the lower the thermal conductivity of the mould material, the longer the cooling time. This effect tends to prolong the overall cycle time, which would affect the (cost) efficiency of production negatively. In this study, the cooling time/cycle time was defined by bending of the sprue such that this hypothesis could not be investigated.

It may be speculated here that replication fidelity on the micro-range and sub-micro-range might even improve due to the lower thermal conductivity of mortar. The lower the thermal conductivity of the (mortar) insert, the longer the time to solidification, which might allow the polymer melt to mimic even deep inner edges and high-aspect-ratio holes before it solidifies.

However, it may be assumed that the thermal conductivity of mortar will increase if a filler material with higher thermal conductivity is selected. This assumption is supported by the fact that concrete mixtures with improved thermal conductivity due to filling with an additive with high thermal conductivity have been described in [16]. The thermal conductivity of alumina at 20 °C is $31.8\frac{W}{m\cdot K}$, which is even higher than the thermal conductivity of Orvar Supreme tool steel, and extensive numeric studies have been carried out to predict the effective thermal conductivity of alumina-containing refractories [17]. The effective thermal conductivity of refractories is influenced by various factors such as grain boundaries, shape of grains and pores and their size distributions, defects such as vacancies, impurities, and isotopes, crystal imperfections such as stacking faults and dislocations, and micro cracks [18].

Consequently, the alumina-containing mortar mixture described in this article might be comparable to Orvar Supreme tool steel rather than to concrete in terms of its effective thermal conductivity. Further investigations need to be carried out in order to reveal the interdependencies between wear rate, replication fidelity, production efficiency (cooling time), effective thermal conductivity, and alumina content.

Part quality is another critical issue that needs to be addressed: (micro) injection moulded polymer parts with constantly high quality require the (mortar) inserts to be temperature-controlled. For small mortar inserts, this may be done by ensuring good contact between the mortar inserts and the temperature-controlled cavity in which they have been fitted. For big mortar inserts, it may be necessary to equip them with cooling channels, which would allow temperature control of the mortar inserts.

One approach may be to use additively manufactured wax channels that would be sunk in the fresh mortar paste upon casting of the mortar insert. After hardening of the mortar paste, the wax could be melted, leaving cooling channels in the mortar insert. This approach might allow incorporating other features in the mortar inserts, for example holes for temperature sensors with inner threads for mounting of these temperature sensors. Additionally, load sensors could be directly incorporated in the mortar paste, which would allow a measurement of internal forces acting in the mortar inserts.

## 5. Conclusions

This paper describes a soft tooling process chain where mortar is used as the final tool insert material. The soft casting template for the mortar can be obtained also by replication or by additive manufacturing. In this paper, a PU cast replica of a milled steel sample was used.

Casting mortar is a completely new rapid tooling approach for micro injection moulding inserts, which we have developed starting from well-known existing concrete/mortar technology. It is possible to cast mortar in soft moulds in order to fabricate hard tools. Therefore, we have found an easy and cheap solution to convert soft moulds or tools into hard moulds or tools.

It has been demonstrated that hard mortar tools can be used as inserts for micro injection moulding purposes. There have not been observed any drawbacks motivated by the brittleness of the material, its lower thermal conductivity, or any wear. Therefore, mortar might be a suitable material, and casting it might be a reliable process for fabrication of injection moulding cavities and inserts for medium to high volume production of polymer (micro) components.
